# Synthesis and Tribological Properties of Bio-Inspired Nacre-Like Composites

**DOI:** 10.3390/ma11091563

**Published:** 2018-08-30

**Authors:** Hang Zhang, Shuhai Liu, Huaping Xiao, Xun Zhang

**Affiliations:** College of Mechanical and Transportation Engineering, China University of Petroleum-Beijing, Beijing 102249, China; zhanghang25@126.com (H.Z.); xz921910@126.com (X.Z.)

**Keywords:** porous ceramics, layered Al_2_O_3_/PMMA composite, freezing casting technology, water-based drilling fluid, abrasive wear

## Abstract

Ceramic materials possessing the properties of high-strength and rigidity are widely used in industry. The shell nacre has a layered structure containing both macroscopic and microscopic levels and is equipped with superior qualities regarding hardness and strength. Therefore, the ceramic composites with a nacre-like layered structure have the potential to be utilized as sliding bearings employed in the harsh conditions of wells. For the purpose of this paper, a porous Al_2_O_3_ ceramics skeleton with nanometer powder is prepared using the freeze-casting method. Then the porous ceramic skeleton is filled with polymer polymethyl methacrylate (PMMA) through mass polymerization to produce a bionic Al_2_O_3_/PMMA composite with a lamellar structure. The properties of the prepared composite are determined by the analysis of micro-hardness, fracture toughness, friction coefficient, wear scar diameter, and the morphology of the worn surface. Consequent results indicate that elevation in the A1_2_O_3_ powder, which acts as the initial solid phase content, prompts the ceramic slurry to exhibit an increase in viscosity and a gradual decrease in the pore size of the ceramic skeleton. The prepared layered Al_2_O_3_/PMMA composite possesses high fracture toughness, which closely resembles that of Al, is approximately four times that of the matrix of the Al_2_O_3_ ceramics and 16 times that of the PMMA. Three kinds of composites containing different solid phase content are subjected to testing involving lubrication by water-based drilling fluid to determine the friction coefficient of each. The results indicate that an increased load leads to a decreased friction coefficient while the impact of speed is not evident. Under dry conditions, the friction coefficient of three different composites tested, declines with elevated load and speed. With the use of water-based drilling fluid as lubrication, the wear scar diameter increases at higher speed, while dry conditions denote increased load. Abrasive wear is determined to be the principal form of erosion of layered Al_2_O_3_/PMMA composites.

## 1. Introduction

Bearings are essential components of downhole tools that are constantly exposed to extreme working conditions [1]. Wear and tear of these bearings is becoming increasingly problematic, severely threatening the operation stability of the bit and other vital equipment [2,3]. As a crucial mechanical component, the ceramic bearing can be widely used in aviation, aerospace, petroleum, automobile, and other fields. Ceramic bearings are unmatched by metal bearings due to their excellent performance at high temperatures, tremendous strength, and outstanding resistance to abrasion and corrosion [4,5,6,7]. However, the notable drawback is that the fracture resilience is exceedingly low. Subsequently, these bearings are prone to microcracks and micro defects, severely restricting its application in engineering [8,9,10]. The hard tissue of natural organisms, especially the nacre of shells, has a lamellar structure from macro to micro [11]. The nacre of the shell is composed of 95 vol. % inorganic aragonite and 5 vol. % organic collagens, which resembles the layered structure of “brick and mud” and can be considered as a type of the ceramic base composite. This bio layered structure material dramatically improves the fracture strength of the matrix material [12,13]. A new method of structure simulation is provided for the preparation of high-performance laminated structure ceramic composites with a shell-like configuration. Moreover, the high performance layered structure material has shown great potential in the field of ion exchange, petrochemical arenas, synthetic materials and more [14]. 

Deville et al. [15,16] prepared hydroxyapatite/epoxy layered structural materials by freeze-casting. The results showed that the best fracture attributes of the shell nacre appeared not only when the inorganic/organic interfacial strength was large, but also when the crack was separated along the inorganic/organic interface before the hard, brittle layer was extended. The typical cracks in these inorganic/organic interfaces transferred to a twisted crack and had a toughening effect on hydroxyapatite/epoxy resin materials, as well as alumina/epoxy resin materials. Surface roughness and unique polymer adhesion control both the mechanical adhesion and chemical adhesion of shell nacre. Thus, high strength and resilience could be obtained. Freeze-casting technology can be widely employed in the application of customized composite materials, such as artificial bone, ceramic/metal composite, bone tissue regeneration, porous scaffold, and so on [17,18,19,20,21,22,23]. Furthermore, the prepared hydroxyapatite layered porous matrix was four times stronger than the porous hydroxyl phosphate stone material prepared using the traditional method. The rigidity of the hydroxyapatite based composite prepared by Deville et al. is 10 GPa, and the strength is 150 MPa, which met the requirements for the use of mineral/organic phase content as 60/40 vol. % high-density bone. 

Ceramic/metal layered composite materials have been studied extensively. The Al-Si/Al_2_O_3_ composite with a solid phase content of 20 vol. % was prepared by Xi Juwei using vacuum-pressure impregnation [24]. The layered structure of the alumina porous ceramic body was replicated, the matrix ceramic layer and the reinforced phase alloy layer were evenly distributed, and the interfacial bonding was satisfactory. The composite exhibited excellent compression strength and fracture toughness. E. Munch et al. used the directional freeze-casting technology to prepare a ceramic suspension in porous ceramics that was then inserted into the polymer to prepare a polymethyl methacrylate (PMMA)/Al_2_O_3_ layered structure composite [25]. The composite material of “brick and mud” with high ceramic content was prepared at the same time. To create a reproduction that closely resembled the microstructure of the pearl layer in the shell, the average lamellar thickness was reduced to 5 μm with the addition of sucrose to the ceramic slurry. The sucrose changed the viscosity and phase diagram of the solvent. The growth of ice crystals produced a micro-roughness on the surface of the ceramic wall equivalent to that of the nacre layer. This structure could be effective in preventing slipping and improving the fracture toughness of materials. Also, the functional groups of methyl methacrylate could be grafted onto the ceramic surface before infiltration of PMMA into the interlayer of ceramic sheets by in situ free radical polymerization. The presence of functional groups of methyl methacrylate could enhance the covalent bond between the two phases. 

Although the study of shell nacre biomimetic ceramic composites has improved the strength, hardness, and toughness of the matrix material to a great extent, and shows excellent development potential, some problems are evident. Presently, the lamellated composite materials are merely parallel structures [26,27]. The freeze-casting device should have the function of cooling both the upper end and the lower end simultaneously, to produce a material possessing a smaller structure exhibiting improved performance. At present, tribological research on shell pearl layer biomimetic laminated ceramic composites is insubstantial. Sliding bearings are critical components of rotary motion. Therefore, the wear problem brought about by a harsh working environment is becoming increasingly noteworthy. Few reports exist regarding the tribological behavior of water-based drilling fluid, bearing material both locally and abroad. The reason for this is the complexity of oil and gas downhole conditions. In this paper, the porous Al_2_O_3_ ceramic preform was prepared by utilizing freeze-casting technology. The prefabricated porous ceramic was filled with PMMA by bulk polymerization, and the ceramic composite with a shell-like structure was prepared. Research into the compression strength, fracture toughness, micro hardness performance, and friction and wear properties, allowed for the material ratio and process parameters to be optimized, providing a wear-resistant sliding bearing composite material of exceptional strength.

## 2. Materials and Methods

### 2.1. Materials

The nano-scale alumina powder (Sigma Aldrich (Shanghai) Trading Co., Ltd., Shanghai, China) used for the purpose of this paper is α-Al_2_O_3_, and its particle size was less than 12 nm. Deionized water was used as the solvent for the freeze-casting experiment. Sodium citrate (Sigma Aldrich (Shanghai) Trading Co., Ltd.) was used as the dispersant. The water-based drilling fluids were prepared for the harsh working conditions like the high water and sand content found in oil/gas wells. The mass ratio of each raw material was low viscosity Carboxyl methyl Cellulose (CMC) (1 wt. %), bentonite (3 wt. %), Na_2_C_3_ (0.15 wt. %), and H_2_O (95.85 wt. %). The sodium carbonate powder was dissolved in the deionized water. The bentonite was added with the glass rod mixing edge, followed by the low viscosity CMC. The mixture was stirred with a magnetic stirrer for 30 min and then left to stand for 24 h.

### 2.2. Experimental Equipment

**Figure 1:**
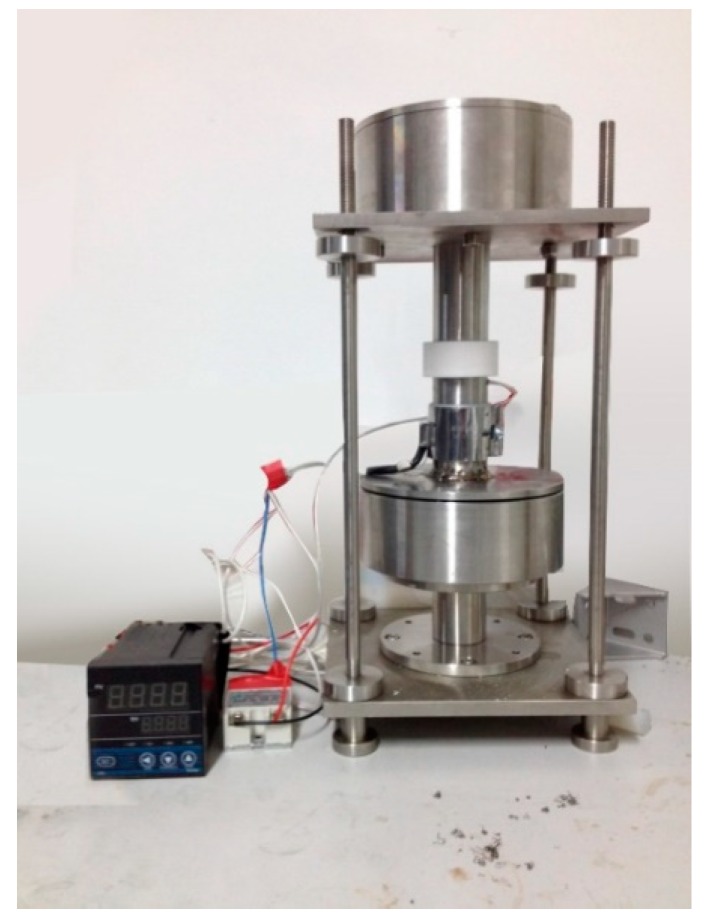
The freeze-casting device.

During the research for this study, a freeze-casting device was designed for directional solidification at the bottom and top of the slurry as shown in Figure 1. The cooling rate at the top and bottom of the slurry could be manipulated with PID (Proportion Integral Derivative) control. It could also be pressurized during the freezing process to achieve a slimmer profile of the lamellar structure and further simulate the nature of the shell nacre. The contact type fast cooling system was designed to apply pressure to the sample at a low temperature and to control the cooling temperature accurately. The freeze-casting device was used to directionally solidify alumina ceramic slurry. It is mainly composed of a refrigeration module, temperature control module, support module and compression module, as illustrated by Figure 1. The experimental platform could simultaneously accomplish, as well as control both the cooling and pressurization of the sample in the refrigeration chamber with remarkable accuracy. A cooling rate of 6 °C/min and a freezing temperature of −30 °C were selected for the experiment. The temperature of the ceramic slurry was reduced from room temperature to −30 °C at a cooling rate of 6 °C/min, and then maintained at −30 °C for 5 min. After the cooling process, a warm insulation procedure was followed to make the cooling more consistent. The detailed experimental equipment in this study is listed in Appendix A.

### 2.3. Preparation of Porous Al_2_O_3_ Ceramics

**Figure 2:**
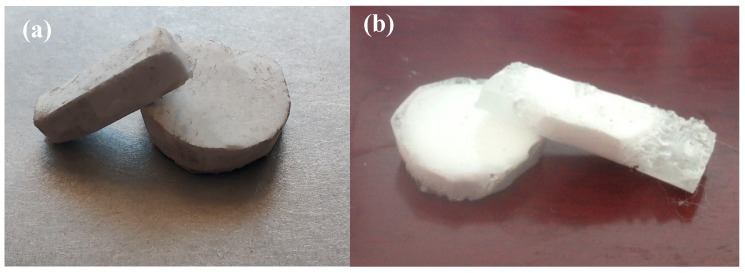
(**a**) Macroscopic morphology of porous alumina; (**b**) Macroscopic morphology of layered Al_2_O_3_/PMMA composite.

The Al_2_O_3_ porous ceramic body was prepared by using freeze-casting technology on ceramic slurry composed of Al_2_O_3_ ceramic powder and deionized water. Three kinds of ceramic pastes with different solid phase contents (the ratio of Al_2_O_3_ powder mass to the entire ceramic slurry) of 15 wt. %, 17.5 wt. %, 20 wt. %, 22.5 wt. %, and 25 wt. %, respectively, were prepared. The total mass of the Al_2_O_3_ ceramic powder was 100 wt. %. The Al_2_O_3_ ceramic powder included 2 wt. % sodium citrate, 1 wt. % polyvinyl alcohol and surplus Al_2_O_3_ nano powder. The prepared slurry was placed into the ball milling tank, followed by further mixing by hand. The slurry was blended for an additional 24 h in the ball milling machine to improve its uniformity and prevent the agglomeration of particles and the combination of flocculation. After the cooling process of the slurry was complete, the sample was placed in the vacuum degasser for 30 min to remove the air and to avoid defects like blowholes. The slurry was deposited into the refrigeration chamber ensuring that the sample was in a vertical temperature gradient environment. This chamber consisted of polytetrafluoroethylene, which produced an excellent thermal insulation effect. The slurry was then subjected to a double-headed direction solidification using a self-made freeze-casting device and was cooled from room temperature to −30 °C. The temperature drop rate was 6 °C/min, and the ceramic slurry was directionally solidified into a cylindrical porous ceramic body of φ25 mm × 5 mm, which was green in color. Following the unmolding of the directionally solidified ceramic body, it was placed in a freeze dryer for 24 h. Low-temperature sublimation was employed to remove the sheet ice from the solidified material to obtain a porous ceramic body. The freeze-dried porous ceramic body was placed in a high-temperature furnace and sintered in nitrogen and then heated to 1650 °C. This temperature was maintained for 4 h, and then allowed to reach room temperature. As shown in Figure 2a, since both the sample size and the content of organic matter are small, no additional organic matter removal was required during the sintering process.

### 2.4. Preparation of Layered Al_2_O_3_/PMMA Composites

The bulk polymerization method was used to insert the PMMA into a porous ceramic body with a solid phase content of 15 wt. %, 17.5 wt. %, 20 wt. %, 22.5 wt. %, and 25 wt. % produced by cryogenic casting technology. The first step was to sand the ceramic body for 20 min using sandpaper with varying grit sizes, followed by a 30 min smoothing process using diamond suspension polishing solution. The sample was then ultrasonically cleaned with petroleum ether and absolute alcohol for 10 min and finally dried. This process was followed by mixing 15 mL of monomeric methyl methacrylate (MMA) (Sigma Aldrich (Shanghai) Trading Co., Ltd., Shanghai, China) and 0.134 g of initiator Azo disobutyronitrile (AIBN) (Accelerating Scientific and Industrial Development thereby Serving Humanity) solution into the dense, layered ceramic body. Prepolymer was prepared by heating it for 30 min–40 min at 70 °C and then leaving it to cool to 40 °C. Finally, the prepolymer was placed in an oven at 40 °C and allowed a reaction time of 24 h in order to obtain the laminated Al_2_O_3_/PMMA composite material, as shown in Figure 2b.

## 3. Micromorphology of Layered Al_2_O_3_/PMMA Composites

Both the prepared porous disc specimens and the layered Al_2_O_3_/PMMA composite disc specimens were sanded using diamond sandpaper with different levels of granularity. A diamond suspension polishing solution was used for further refinement, followed by an ultrasonic cleaning with petroleum ether and anhydrous ethanol. Finally, the specimen was dried, and the spray gold was produced. The interface microstructure of porous ceramics and layered Al_2_O_3_/PMMA composites were observed and analyzed by an SU8010 Cold Field Emission Scanning Electron Microscope (Hitachi (China) Co., Ltd., Beijing, China).

**Figure 3:**
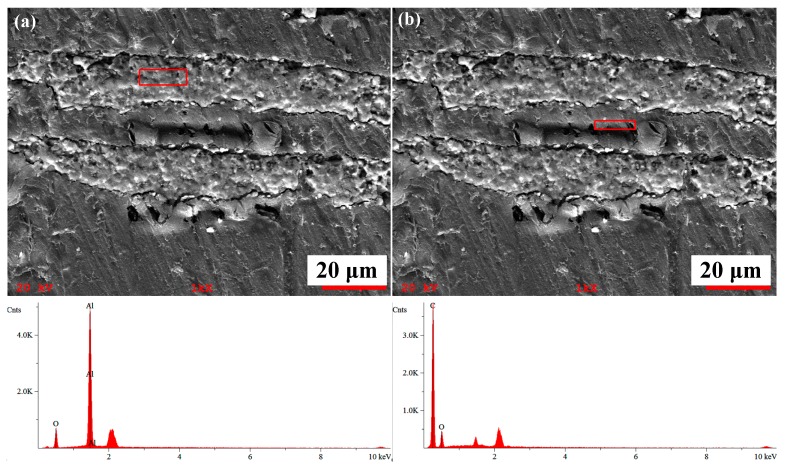
EDS (Energy Dispersive Spectrometer) analysis of laminated Al_2_O_3_/PMMA composites: (**a**) Al_2_O_3_ region; (**b**) PMMA region.

Figure 3 shows the EDS (Energy Dispersive Spectrometer) analysis of laminated Al_2_O_3_/PMMA composites. The results show that after the porous ceramic body was filled with polymer PMMA, both the ceramic and the polymer interface displayed proper integration. The improved layered structure was established, indicating that the bulk polymerization process was satisfactory and the prepared Al_2_O_3_/PMMA composite possessed an adequate stratified form. In Figure 3a, the O atomic content in the red box area is 42.02 wt. % and the Al atomic content is 57.98 wt. %, which confirms that the area consists of matrix material Al_2_O_3_. Figure 3b shows that the C atomic content in the red box area is 71.19 wt. % and the O atomic content is 28.82 wt. %, which indicates that the area is comprised of filled phase PMMA.

**Figure 4:**
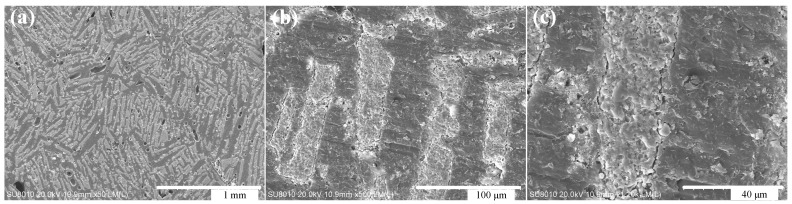
(**a**–**c**) Microscopic morphology of 20% wt. % layered Al_2_O_3_/PMMA composite with different magnification.

**Figure 5:**
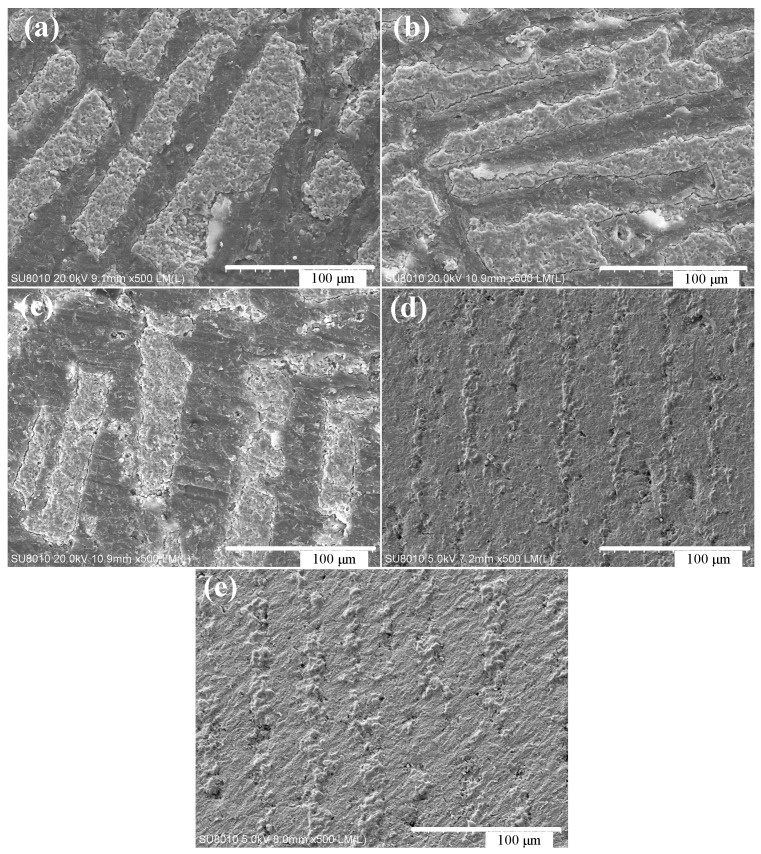
Microscopic morphology of the cross-section of layered Al_2_O_3_/PMMA composites with different initial solid content (freezing temperature is 30 °C): (**a**–**e**) 15 wt. %, 17.5 wt. %, 20 wt. %, 22.5 wt. %, and 25 wt. %.

Figure 4 shows the microstructure of layered Al_2_O_3_/PMMA composites with an initial solid content of 20 wt. % prepared by freeze-casting and bulk polymerization. It is clear that the ceramic layer (bright color on the map) and the polymer layer (the dark color) are distributed alternately, and the porous structure of the alumina is inherited. The interface of both the matrix alumina and the reinforcing phase polymer PMMA is satisfactory denoting the adequacy of the bulk polymerization process. Figure 5a illustrates the random nature of the pore orientation in the cross-section of the sample. The reason for this pertains to the direction the formation and growth of ice crystals take along the path of the temperature gradient, which lies perpendicular to the cross-section of the sample. A number of small holes are evident in the polymer PMMA layer of the sample, which is the result of the higher temperature of the bulk polymerization process that leads to the formation of bubbles in the moulded PMMA. Therefore, the temperature of the prepolymerization and polymerization stage of the bulk polymerization process should be strictly controlled to prevent the product from producing bubbles that can influence the performance of the sample. After bulk polymerization the laminar structure of the composite is more pronounced. Both the matrix ceramics and the reinforcing phase PMMA are well combined, and the microstructure is more compact.

Figure 5 shows the microscopic morphology of the layered Al_2_O_3_/PMMA composites with varying initial solid content as exposed to a temperature of −30°C. The layered Al_2_O_3_/PMMA composites prepared by cryopreservation and bulk polymerization have a micro morphology of ceramic lamellae, and polymer PMMA lamellae arranged alternately. As the initial solid phase content increases, so does the thickness of the sample’s ceramic layer until it reaches approximately 20–30 μm in both the ceramic and polymer layers. The lamellar structure is thinner and closer in appearance to the nanoscale layer structure of the shell nacre. This is an indication that the double-headed freeze method is able to refine the composite layer structure. The bulk polymerization process does not destroy the layered pore structure of the porous ceramic body. The bulk polymerization process allows the PMMA to be adequately inserted into the layered pore structure of the billet, replacing the region of the ice crystals before the introduction of sublimation during the freeze-casting process. This encourages the formation of both the laminated structure of the ceramic layer and the PMMA layer. Therefore, upon completion of the bulk polymerization process, the microscopic morphology of the composite material inherits the layered structure of the porous ceramic billet, as well as the microscopic morphology of the ceramic body containing lamellar pores.

## 4. Properties of Layered Al_2_O_3_/PMMA Composites

### 4.1. Fracture Toughness

**Figure 6:**
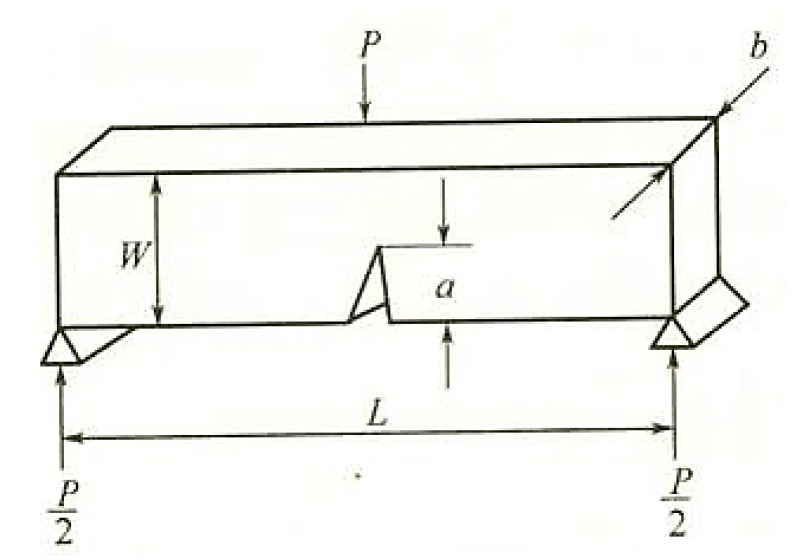
Shape, size, and stress diagram of Chevron Notch (CN) sample.

The fracture toughness of laminated Al_2_O_3_/PMMA composites was measured by the Chevron Notch (CN) method. Figure 6 depicts the shape, size, and stress diagram of the CN sample. The size of the laminated Al_2_O_3_/PMMA composite specimen is (L × W × B), 20 mm × 4 mm × 7 mm, the incision depth is 1 mm, and the distance between the two supporting points below is 16 mm. A universal electronic material testing machine was used to measure the maximum load, as represented by P, of the layered Al_2_O_3_/PMMA composites containing diverse solid phase content, as shown in Table 1.

The formula for the determination of fracture toughness by CN method is:

(1) $$K_{IC}=P/\left(b\sqrt{W}\right)\left[17.99+67.43\left(\frac{a}{w}\right)+69.65{\left(\frac{a}{W}\right)}^{2}+72.07{\left(\frac{a}{W}\right)}^{3}+636.8{\left(\frac{a}{W}\right)}^{4}\right]$$

where *P* is load (N), *W* is sample height (m), *b* is sample width (mm), *a* is incision depth (m), and *K_IC_* is fracture toughness (MPa·m^1/2^).

The fracture toughness of layered Al_2_O_3_/PMMA composites with different solid phase contents calculated is shown in Table 2.

According to existing literature, the fracture toughness of the matrix Al_2_O_3_ ceramic is 3–5 MPa·m^1/2^, the fracture toughness of the reinforcing phase polymer PMMA is 0.7–1.6 MPa·m^1/2^, and the fracture toughness of the metal Al is 14–28 MPa·m^1/2^. Therefore, the prepared layered Al_2_O_3_/PMMA composite has excellent fracture toughness, which significantly improves the fragility of the ceramic matrix, and can effectively improve the performance of the ceramic sliding bearing. Here, the crack deflection model is referred to by way of explanation. Deflection will occur when a crack is extended in both the flexible (polymer PMMA) and brittle (Al_2_O_3_ ceramic) layer, exhibiting Al_2_O_3_ as a staircase distribution. The ductile layer can absorb energy through plastic deformation, and when it is compressed, it can form macro bridging at the tail of a crack to prevent it from spreading, thus effectively improving the fracture toughness of the material.

### 4.2. Microhardness

**Figure 7:**
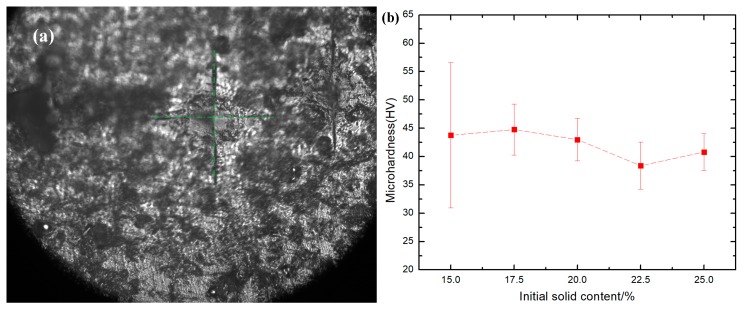
(**a**) Surface indentation of layered Al_2_O_3_/PMMA composites; (**b**) Microhardness of layered Al_2_O_3_/PMMA composites with different initial solid phase content.

**Figure 8:**
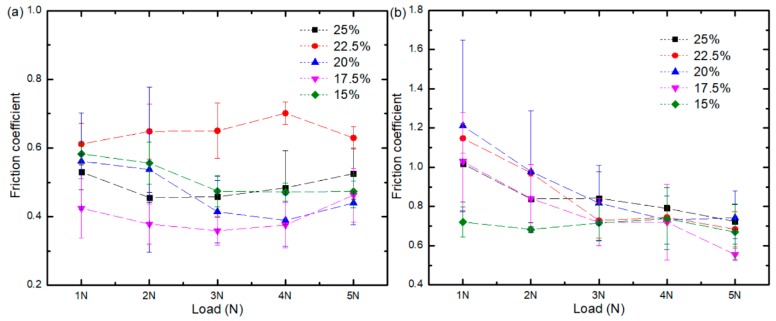
Variation curves of the friction coefficient of composites with different solid phase content: (**a**) Water-based drilling fluid; (**b**) Dry friction.

The prepared layered Al_2_O_3_/PMMA composite disc specimen was polished using sandpaper with varying particle sizes of diamond and diamond suspension polishing solution. The polishing process was followed by an ultrasonic wash with petroleum ether and absolute ethanol, and finally dried. The HVT-1000Z Microhardness Analyzer (Shanghai Zhongyan Instrument Factory, Shanghai, China) was used to measure the surface microhardness of the layered Al_2_O_3_/PMMA composite. A study was made of changes occurring in the microhardness of the sample with the solid phase content with a load of 2.942 N and a loading time of 10 s. Ten measurements were taken, and the error margin determined to arrive at the final result. As shown in Figure 7a, the surface indentation of laminated Al_2_O_3_/PMMA composites was observed by a microhardness analyzer. The indentation is diamond shaped and appears to be cracking, indicating that the material is fairly brittle. Figure 8b shows the microhardness of layered Al_2_O_3_/PMMA composites with solid phase content. It is evident that the microhardness of layered Al_2_O_3_/PMMA composites decreases in conjunction with the increase of initial solid phase content. The levels of microhardness of the composites 15 wt. %, 17.5 wt. %, 20 wt. %, and 22.5 wt. %, and 25 wt. %, are HV 43.75, HV 44.77, HV 42.99, HV 38.39, and HV 40.81, respectively. The higher the content of polymer PMMA in layered Al_2_O_3_/PMMA composites, the less plastic deformation is produced. Thus, the likelihood of the indentation being generated is markedly reduced. With the microhardness value fluctuating between 44 HV and 38 HV, it is generally less effective within the solid concentration range (15–25 wt. %) used in the experiment.

### 4.3. Sliding Friction Characteristics of Layered Al_2_O_3_/PMMA Composites

The friction coefficient of the layered Al_2_O_3_/PMMA composites with different solid content was studied by applying variable levels of loading and rotational speed. The correlating friction and wear test was executed by the CFT-1 material surface performance tester. The initial method of interaction in the friction and wear experiment was point contact when the ball-disk sample was adopted. The disk assumes the cylindrical sample of solid phase content of 15 wt. %, 17.5 wt. %, 20 wt. %, 22.5 wt. %, and 25 wt. %, respectively, as well as a Q235 standard steel ball of φ4 mm. The content of main alloying elements of the steel ball are: C (0.14%–0.22%); Mn (0.30%–0.65%); Si (≤0.30%); S (≤0.04%); and P (≤0.04%). The rotational speeds set in the experiment were 100 r/min, 150 r/min, 200 r/min, 250 r/min, and 300 r/min, with the loads at 1 N, 2 N, 3 N, 4 N, and 5 N, respectively. The experimentation time was 10 min, and the experimental environment was kept at room temperature. The samples were either immersed in the water-based drilling fluid or tested under dry friction conditions. The VKX-100 Shape Measurement Maser Microscope (Keyence (China) Co., Ltd., Shanghai, China) provided by was employed to observe and measure the wear scar diameter of laminated Al_2_O_3_/PMMA composites.

Figure 8 shows the variation curve of the friction coefficient of water-based drilling fluid and dry friction material at different initial solid phase content with loads at a speed of 200 r/min. Figure 8a, illustrates that the friction coefficient of composites at 15 wt.%, 17.5 wt.%, 20 wt.%, and 22.5 wt.% is generally reduced when the load is increased. The friction coefficient ranges generated using water-based drilling fluid are as follows: for the 15 wt. % composite it is between 0.47 and 0.58; for the 17.5 wt. % composite it is between 0.35 and 0.46; for the 20 wt. % composite it is between 0.37 and 0.56; for the 22.5 wt. % composite it is between 0.61 and 0.70; and for the 25 wt. % composite it is between 0.46 and 0.53. From Figure 8b it is clear that the friction coefficient of composites with different initial solid phase content decreased with a higher load when tested under dry conditions. As a result, the more extensive the load, the more ceramic solid particles will be formed by the grinding of ceramic and steel balls. Consequently, the rolling effect is more apparent, and the friction coefficient becomes smaller. The friction coefficient ranges generated in dry conditions are as follows: for the 15 wt. % composite it is between 0.66 and 0.73; for the 17.5 wt. % composite it is between 0.55 and 1.03; for the 20 wt. % composite it is between 0.73 and 1.21; for the 22.5 wt. % composite it is between 0.68 and 1.15; and for the 25 wt. % composite it is between 0.72 and 1.12. The friction coefficient of the 15 wt.% composites displayed little variation, while the friction coefficient of 17.5 wt.%, 20 wt.%, 22.5 wt.%, and 25 wt.% composites exhibited considerable diversity. Compared with the water-based drilling fluid environment, under dry conditions, no lubrication is present to act as a barrier between the friction pairs. Therefore, the two friction surfaces come into direct contact with each other leading to a larger unstable friction coefficient. Also, the friction coefficient increases with the increase of solid phase content. When the steel ball and the alumina are in contact with each other, elastic or plastic deformation is likely to occur. The higher the initial solid phase content, the higher the ceramic content, the higher the chance of the steel ball and the ceramic grinding, the larger the contact stress, and the larger the frictional resistance.

**Figure 9:**
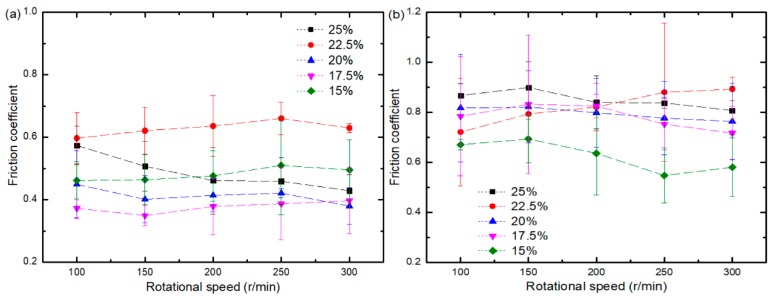
The friction coefficient of composites with different initial solid phase content at different speeds: (**a**) Water-based drilling fluid; (**b**) Dry friction.

Figure 9 shows the variation curve of the friction coefficient of the composite as tested in both a lubricated and dry environment containing different initial solid phase content with a load of 3 N. Figure 9a indicates that changes in the friction coefficient of five kinds of composites with different solid phase content are not evident following the application of rotational speed. The friction coefficient ranges are as follows: for the 15 wt. % composite it is between 0.46 and 0.50; for the 17.5 wt. % composite it is between 0.34 and 0.49; for the 20 wt. % composite it is between 0.37 and 0.44; for the 22.5 wt. % composite it is between 0.60 and 0.66; and for the 25 wt. % composite it is between 0.43 and 0.57. From Figure 9a, it is clear that the friction coefficient displays no apparent upward or downward trend with the increase in velocity but is stable instead. This result is due to the low viscosity of the water-based drilling fluid, which makes it difficult to form fluid dynamic lubrication. Mixed lubrication still dominates the lubrication state, and all the friction coefficients are stable. Figure 9b illustrates that the friction coefficient of the five solid phase content composites decrease when rotational speed is higher. Due to the low microhardness of these composites, the ceramic solid particles that detach under the load weight produce a rolling effect and the friction coefficient of the composite decreases. 

Contrary to the results obtained from testing conducted in the lubricated environment, the friction coefficient is unmistakably higher under dry conditions due to the lack of lubrication by water-based drilling fluids. The friction coefficient gradually increases with the elevation of the solid phase content. This elevation increases the ceramic content in the composite, reinforcing the probability of the ceramic and the steel ball making abrasive contact. The possibility increases for elastic or plastic deformation to occur hindering sliding of the bearing. Thus, the friction coefficient is higher.

### 4.4. Wear Characteristics of Layered Al_2_O_3_/PMMA Composites

**Figure 10:**
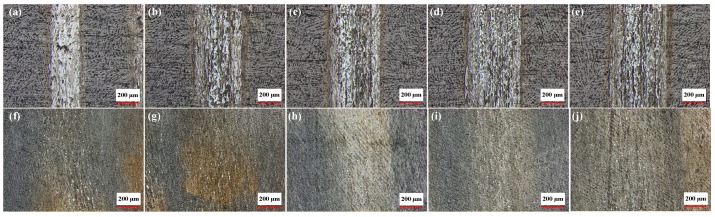
Wear scar morphology of 25 wt. % initial solids phase content composites at 3 N under dry friction conditions with speed of: (**a**) 100 r/min; (**b**)150 r/min; (**c**) 200 r/min; (**d**) 250 r/min; and (**e**) 300 r/min. Wear scar morphology of 25 wt. % initial solids phase content composites at 3N under water-based drilling fluid conditions with speed of: (**f**) 100 r/min; (**g**) 150 r/min; (**h**) 200 r/min; (**i**) 250 r/min; and (**j**) 300 r/min.

**Figure 11:**
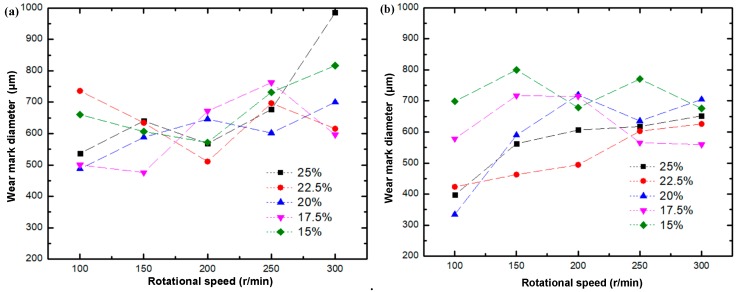
Change curve of wear scar diameter of composites of different initial phase solids content with rotational speed: (**a**) Water-based drilling fluid; (**b**) Dry friction.

Figure 10 shows the surface morphology of the 25 wt. % initial solid phase content composite subjected to testing in both a dry environment and in the presence of water-based drilling fluid with a fixed load of 3 N, and speeds of 100 r/min, 150 r/min, 200 r/min, 250 r/min, and 300 r/min, respectively. The wear scar of the composite material in the water-based drilling fluid is more pronounced. Figure 11 shows the change curve of the width of the wear scar on the Al_2_O_3_/PMMA composite comprised of varying solid phase content. When water-based drilling fluid is used, the width of the wear scar displays a general increase at a higher rotational speed.

**Figure 12:**
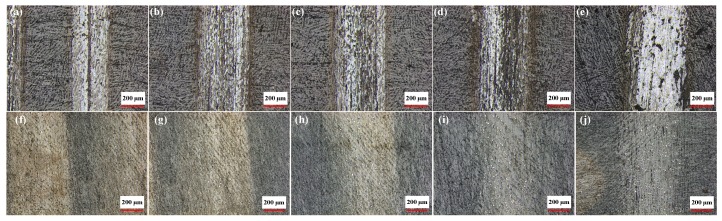
Wear scar morphology of 25 wt. % initial solid phase content composites with a rotational speed of 200 r/min under dry friction conditions: (**a**) load:1 N; (**b**) Load: 2 N; (**c**) Load: 3 N; (**d**) Load: 4 N; and (**e**) load: 5 N. Wear scar morphology of 25 wt.% initial solid phase content composites with a rotational speed of 200 r/min under water-based drilling fluid conditions: (**f**) Load: 1 N; (**g**) Load: 2 N; (**h**) Load: 3 N; (**i**) Load: 4 N; and (**j**) load: 5 N.

**Figure 13:**
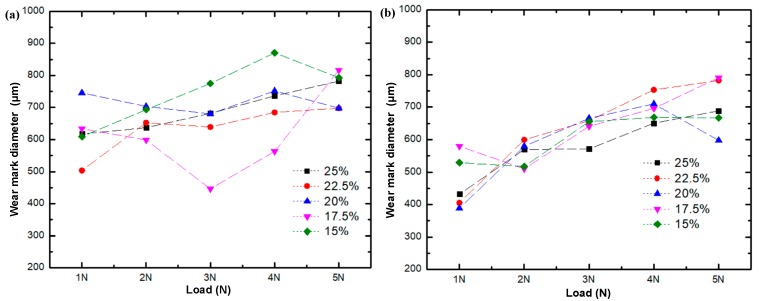
Variation of wear scar diameter of composites of different initial phase solid content with the load under (**a**) water-based drilling fluid; (**b**) dry friction conditions.

Figure 12 illustrates the surface morphology of the 25 wt. % initial solid phase content composite, representative of testing conducted in both lubricated and dry conditions at a fixed speed of 200 r/min, with respective loads of 1 N, 2 N, 3 N, 4 N, and 5 N. Figure 13 shows the change curve of the diameter of the wear scar on the layered Al_2_O_3_/PMMA composites comprised of different solid phase content. The wear scars of five kinds of initial solid phase content composites were tested and were found to widen under increased load weight in both lubricated and dry conditions. The elevated load is responsible for a gradual increase in the number of corrosion pits and furrows on the surface of the steel ball leading to abrasive and corrosion wear. Therefore, the friction coefficient is higher.

**Figure 14:**
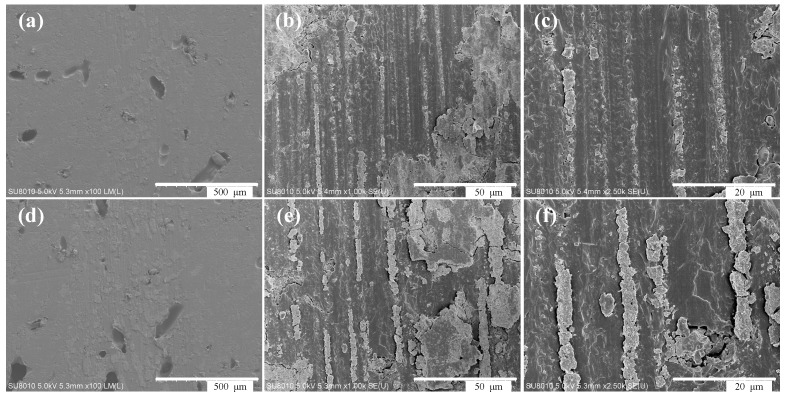
(**a**–**c**) Surface wear morphology of composites under dry friction at 1 N, 200 r/min; and (**d**–**f**) 3 N, 300 r/min with different magnification.

Figure 14 shows the wear morphology of the composite surface under dry friction conditions. Figure 14a–c indicates that when the load is 1 N, and the speed is 200 r/min, the eroded surface of the composite material with an initial solid phase content of 20 wt. % is furrowed, and the wear form is mainly abrasive. As shown in Figure 14d–f, when the load is 3 N, and the speed is 300 r/min, the worn surface of the composite material with an initial solid phase content of 20 wt. % becomes more apparent, while the wear form is mainly abrasive.

## 5. Conclusions

Freeze-casting technology was used in conjunction with nano-scale Al_2_O_3_ powder, to successfully prepare a porous ceramic body with an unmistakable lamellar structure after sintering at 1650 °C for four hours. With the increase in initial solid phase content, the viscosity of the ceramic slurry increased, while the pore size decreased. The selected composite material represented by 15 wt. %, 17.5 wt. %, 20 wt. %, 22.5 wt. %, and 25 wt.%, respectively, had individual pore sizes of 24, 18, 16, 15, and 10 μm. The respective thicknesses of the ceramic sheets were 22, 30, 32, 15, and 20 μm, with a slimmer lamellar structure. The method of bulk polymerization was used to insert the polymer PMMA into the porous ceramic body to produce a layered Al_2_O_3_/PMMA composite with a suitable combination of matrix ceramics and enhanced phase polymers. The thickness of both the ceramic layer and the polymer layer was approximately 20–30 μm, and the lamellar structure was thinner. The prepared composites had high fracture toughness closely resembling that of Al, about four times that of matrix Al_2_O_3_ ceramics and 16 times that of enhancement phase PMMA. When using water-based drilling fluid on five kinds of composites containing different solid phase content, the friction coefficient decreased under a heavier load. No change was apparent with the application of speed. The friction coefficient of three different solid phase content composites was tested in a dry friction environment and was found to follow a downward trend with the increase of both load and rotational speed. Exposure to lubricated conditions and elevated rotational speed lead to the expansion of the wear scar diameter. Testing conducted in dry conditions showed an identical result when the load was increased. The synthetic nacre-like composites in this study can contribute to a wear-resistant sliding bearing composite material of exceptional strength in petroleum drilling equipment.

## Figures and Tables

**Table 1:** Maximum load of layered Al_2_O_3_/PMMA composites with different initial solid phase content.

Initial solid phase content	15%	17.5%	20%	22.5%	25%
Maximum load (N)	328.492	176.176	247.942	211.852	297.768

**Table 2:** Fracture toughness of layered Al_2_O_3_/PMMA composites with different initial solid phase content.

Initial solid phase content	15%	17.5%	20%	22.5%	25%
Fracture toughness (MPa·m^1/2^)	31.7695	17.038	23.978	20.488	28.797

## Abbreviations

PMMA	polymethyl methacrylate
CMC	Carboxyl methyl Cellulose
MMA	monomeric methyl methacrylate
AIBN	Azo disobutyronitrile
CN	Chevron Notch
PID	Proportion Integral Derivative

## Appendix A

Experimental Apparatuses

**Table A1:** Experiment apparatuses.

Instruments	Model	Manufacturer	Usage
Electronic balance	BS224S	Sartorius-mechatronics (Beijing) Co., Ltd., Beijing, China	Weighing
Ball mill	SFM-3	Hefei kejing materials technology Co., Ltd., Hefei, China	Slurry preparation
Rotary-vane Vaccum Pump	VP-1	Taizhou zhengkong pump industry Co., Ltd., Taizhou, China	Slurry degassing
Freeze-casting device	——	Self-made, Beijing, China	Directional solidification of slurry
Freeze drier	FD-1A-50	Shanghai bilang manufacturing Co., Ltd., Shanghai, China	Cryogenic distillation
Muffle furnace	KSL-1700X	Hefei kejing materials technology Co., Ltd., Hefei, China	Ceramic sinter
Water bath kettle	XMTD-204	Chunlan experimental instrument factory, Changzhou, China	Prepolymerization
Oven	ZK-30AB	Shanghai shenguang instrument and meter Co., Ltd., Shanghai, China	polymerization
Grinding and polishing machine	UNIPOL-1200S	Shenyang kejing automation equipment Co., Ltd., Shenyang, China	Polishing
Ultrasonic cleaner	KX-1840T	Manufacture Expert, Tianjin, China	Cleaning the sample
Bench diamond wire cutter	STX-402	Shenyang kejing automation equipment Co., Ltd., Shenyang, China	Cutting sample
Universal electronic material testing machine	LN-939CS	Guangdong lina industrial Co., Ltd., Guangdong, China	Determine sample compression strength
Cold field emission scanning electron microscope.	SU8010	Hitachi (China) Co., Ltd., Beijing, China	Observe the interface microstructure of porous ceramics and layered composites
Microhardness analyzer	HVT-1000	Shanghai zhong yan instrument factory, Shanghai, China	Measure the surface microhardness of the layered composite.
Material surface performance tester.	CFT-1	Lanzhou zhongke kaihua technology Co., Ltd., Lanzhou, China	The correlating friction and wear test
Laser microscope	VKX-100	KEYENCE (China) Co., Ltd., Shanghai, China	Measurement of roughness and worn marks diameter
